# Book Reviews

**Published:** 1913-12

**Authors:** 


### BOOK REVIEWS

OBSTETRICS. A Manual for Students and Practitioners. By W. P. Manton, M. D., Professor of Obstetrics and Clinical Gynecology, Detroit College of Medicine, Detroit, Mich. Second edition, revised and enlarged; including selected list of State Board Examination Questions. 12mo, 292 pages, with 97 engravings. Cloth,

$1.00, net. Lea & Febiger, Publishers, Philadelphia and New York, 1913.

This little volume fills admirably the twofold purpose for which it was crated, namely, a convenient manual by which the physician can quickly refresh his memory, and an excellent means by which the student can review his course on obstetrics in preparing for examination. The questions appended to each chapter will be found a strong mental stimulus. The revision for this new edition has been so thorough that it has amounted virtually to a rewriting, so that the book is really a new one. It is exceptionally well illustrated, and is typographically all that should be desired. Its large circulation is apparent in the unusual value which the purchaser receives.

---

HOW TO COLLECT A DOCTOR BILL. By Frank P. Davis, M. D. Published by author. Price $1.00. Enid, Okla.

Davis directs attention to a most important phase of the physician's work and duty. Every physician reads and follows the author's suggestions will soon collect, in addition to his usual collections, vastly more than the price of this useful little book.

---

MALARIA: Etiology, Pathology, Diagnosis, Prophylaxis, and Treatment. By Graham E. Henson, M. D., with an introduction by Chas. E. Bass, M. D. Twenty-seven illustrations. Pp. 190. Price $2:50. Published by C. V. Mosby Co., St. Louis, Mo.

The importance of this subject cannot be overestimated and it is of peculiar timeliness at present when our relations with tropical and sub-tropical countries are to increase with completion of the Planama Canal. This is a most valuable monograph and we highly commend it to our readers.

